# The optimal discovery procedure for significance analysis of general gene expression studies

**DOI:** 10.1093/bioinformatics/btaa707

**Published:** 2020-08-20

**Authors:** Andrew J Bass, John D Storey

**Affiliations:** Lewis-Sigler Institute for Integrative Genomics, Princeton University, Princeton, NJ 08544, USA; Lewis-Sigler Institute for Integrative Genomics, Princeton University, Princeton, NJ 08544, USA

## Abstract

**Motivation:**

Analysis of biological data often involves the simultaneous testing of thousands of genes. This requires two key steps: the ranking of genes and the selection of important genes based on a significance threshold. One such testing procedure, called the optimal discovery procedure (ODP), leverages information across different tests to provide an optimal ranking of genes. This approach can lead to substantial improvements in statistical power compared to other methods. However, current applications of the ODP have only been established for simple study designs using microarray technology. Here, we extend this work to the analysis of complex study designs and RNA-sequencing studies.

**Results:**

We apply our extended framework to a static RNA-sequencing study, a longitudinal study, an independent sampling time-series study,and an independent sampling dose–response study. Our method shows improved performance compared to other testing procedures, finding more differentially expressed genes and increasing power for enrichment analysis. Thus, the extended ODP enables a favorable significance analysis of genome-wide gene expression studies.

**Availability and implementation:**

The algorithm is implemented in our freely available R package called edge and can be downloaded at https://www.bioconductor.org/packages/release/bioc/html/edge.html.

**Supplementary information:**

Supplementary data are available at *Bioinformatics* online.

## 1 Introduction

Genome-wide gene expression studies measure simultaneously the expression levels of thousands of genes using RNA-seq or DNA microarray technology. A primary objective in these studies is to discover biologically important genes by applying appropriate statistical tools to the data. One such approach is to apply a hypothesis testing procedure on a gene-by-gene basis to detect differentially expressed genes; for example, a *t*-test or *F*-test is commonly used to compare multiple biological groups. These test statistics are then ranked and a subset of genes with values above a specified threshold are deemed statistically significant. The significance threshold is chosen to control the false discovery rate (FDR), which is the expected proportion of false positives in the subset of selected genes. Thus, there are two key steps when selecting important genes: the ranking of test statistics and the selection of tests based on a significance threshold.

One commonly used class of testing procedures to rank genes is based on the likelihood ratio test (LRT). The LRT compares the goodness-of-fit between two models, namely, the alternative and null models. The test statistic is the ratio of the likelihood under the alternative model to the likelihood under the null model, where large values indicate evidence against the null model. It is popular due to its optimality guarantees: the Neyman–Pearson (NP) lemma states that the LRT statistic is the most powerful testing procedure for a single hypothesis, where no other testing procedure can achieve more power at a fixed significance threshold (Neyman and Pearson, 1933). However, the LRT statistic may not provide the optimal ranking for multiple hypotheses (Storey, 2007). This is problematic in genomics where thousands of tests are typically performed.

When there are multiple hypotheses, such as in gene expression data, many testing procedures can improve upon the statistical power using information across genes. One such method is the optimal discovery procedure (ODP), which maximizes the number of expected true positives for a fixed number of expected false positives—a quantity related to the FDR (Storey, 2007). The ODP is a generalization of the NP lemma: while the NP lemma is optimal for a *single* hypothesis test, the ODP is optimal for *multiple* hypothesis tests. The ODP achieves the optimal ranking of test statistics by leveraging information across all tests when calculating the test statistic for each gene. Intuitively, the improvement in ranking stems from genes that follow similar patterns of expression (known as co-expression). This information is incorporated into the test statistic to quantify the evidence for differential expression. In the study by Storey *et al.* (2007), an approximation to the ODP performs favorably on DNA microarray studies compared to SAM (Tusher *et al.*, 2001), shrunken *t*-test or *F*-test (Cui *et al.*, 2005; Smyth, 2004), Bayesian local FDR (Efron *et al.*, 2001) and posterior probabilities (Lönnstedt and Speed, 2002).

There are two main limitations when applying the ODP to genomic studies. First, the method was primarily developed for simple static experiments (e.g., comparing two conditions) and it has not yet been extended to more complex sampling designs. Second, the underlying assumption in the ODP is that the data are generated from a Normal distribution where the per-gene observations have the same variance (i.e., homoscedasticity). This is problematic for RNA-sequencing studies where the data are modeled using an over-dispersed Poisson distribution or a Normal distribution where the per-gene observations have different variances (i.e., hetereoscedasticity) (Law *et al.*, 2014). Due to these constraints, the applicability of the ODP has been limited to static DNA microarray studies.

In this work, we extend the ODP to both complex study designs and RNA-sequencing studies. To incorporate non-linear responses commonly found in non-static studies, we utilize the regression spline methodology from Storey *et al.* (2005). An advantage of this approach is that it flexibly models gene expression responses within the least squares framework where the data are assumed to follow a Normal distribution with constant variance: this enables for a straightforward application of the ODP. For RNA-sequencing studies, we implement the same strategy in Law *et al.* (2014) to estimate the per-gene hetereoscedasticity using the observed mean–variance relationship. We then use these estimated weights in a weighted least squares algorithm to adjust for unequal variances among observations. This transformation allows for the standard ODP framework to be utilized.

We apply our algorithm to three different experimental designs. The first is a ‘static sampling’ experiment, where samples are obtained at a fixed time point. For this example, we analyze a smoker study where smoking and non-smoking groups are compared to detect transcriptional differences in airway basal cells using RNA-seq technology. The second is an ‘independent sampling’ experiment, where subjects are independently sampled across time or dosage level. Here, we consider two independent sampling studies, namely, a time-series and a dose–response study. The former considers the effect of age on gene expression in the cortex region of the kidney and the latter explores breast cancer cell sensitivity in response to multiple 17*β*-estradiol doses. The final design is a ‘longitudinal sampling’ experiment, where subjects are sampled at multiple time points. As an example, we examine an endotoxin study which compares the leukocytes at a control group to those of an endotoxin-treated group across multiple time points.

The article is outlined as follows. Section 2 reviews background on the ODP and regression splines. We also review a computationally efficient implementation of the ODP called the modular optimal discovery procedure (mODP). Section 3 introduces our proposed algorithm, and Section 4 illustrates the results from our method on the four studies. We validate these results through comprehensive simulations.

## 2 Background

### 2.1 The optimal discovery procedure

The optimal (or ‘most powerful’) hypothesis test statistic for a single test is provided by the NP lemma (Neyman and Pearson, 1933). Given some observed data $y=(y_{1},y_{2},\ldots ,y_{n})$, the NP lemma states that the ratio of the alternative likelihood g(y) over the null likelihood f(y)—known as the likelihood ratio—has the largest power for each false-positive rate compared to any other test statistic. Intuitively, this optimality arises because the data-generating process under each model is assumed to be known. For multiple hypotheses, the likelihood ratio could be applied on a test-by-test basis. However, potentially useful information across different hypotheses is ignored. As a consequence, the NP likelihood ratio test statistic may no longer be an optimal statistical test (Storey, 2007).

The ODP is a generalization of the NP lemma for multiple hypotheses. More specifically, consider gene expression measurements $\left(y_{1},y_{2},\ldots ,y_{m}\right)$ where there are *m* genes and *n* observations. Further assume that the first *m*_0_ and the last $m_{1}=m-m_{0}$ hypotheses are from the null and alternative models, respectively. The ODP test statistic for gene *i* is

(1) $$S_{\text{ODP}}(y_{i})=\frac{\sum_{k=m_{0}+1}^{m}g_{k}(y_{i})}{\sum_{k=1}^{m_{0}}f_{k}(y_{i})},$$

where $g_{k}(\cdot )$ is the alternative likelihood and $f_{k}(\cdot )$ is the null likelihood for gene *k*. The numerator and denominator can be viewed as the cumulative likelihoods under the alternative and null models, respectively, across all hypotheses , where the likelihoods most related to gene *i* contribute the most to the above statistic. Storey (2007) shows that this test statistic maximizes the number of expected true positives (ETP) for a fixed number of expected false positives (EFP)—a quantity closely related to the FDR, in that $\text{FDR}\approx \frac{\text{EFP}}{\text{EFP}+\text{ETP}}$. Therefore, by leveraging information across different hypotheses, the ODP achieves the optimal significance ranking of test statistics. Moreover, the improvements in statistical power are sometimes substantial compared to the standard likelihood ratio test based on the NP lemma (Storey, 2007).

Evaluating equation (1) requires making assumptions on the data-generating process and the hypothesis status for each test. In this work, the alternative and null densities follow a Normal distribution with some mean vector $\mu =(\mu_{1},\mu_{2},\ldots ,\mu_{n})$ and standard deviation *σ*, denoted by $\left(\mu_{i}^{1},\sigma_{i}^{1}\right)$ and $\left(\mu_{i}^{0},\sigma_{i}^{0}\right)$ for genes i=1,2,…,m, respectively. However, it is not known *a priori* which tests are from the alternative and null models. Instead an approximation to the true ODP statistic is estimated (Storey *et al.*, 2007), i.e., ${\hat{S}}_{\text{ODP}}(y_{i})=\frac{\sum_{k=1}^{m}g_{k}(y_{i};\mu_{k}^{1},\sigma_{k}^{1})}{\sum_{k=1}^{m}f_{k}(y_{i};\mu_{k}^{0},\sigma_{k}^{0})}$. Another complication is that the theoretical null distribution of the ODP test statistic is unknown and so *P*-values cannot be analytically calculated. Therefore, a bootstrap procedure must be implemented to generate an empirical null distribution of the test statistics. This estimated ODP has been shown to provide similar power to the true ODP (Storey *et al.*, 2007). However, calculating the test statistics involves $2m^{2}$ computations and so it is computationally demanding for genomic datasets where *m* can range anywhere from 10^3^ to 10^5^.

Woo *et al.* (2011) proposed a computationally efficient approximation to the ODP called the modular ODP (mODP). Similar to the estimated ODP, the mODP assumes that the data $y_{i}$ are generated from a Normal distribution with parameters $\left(\mu_{i}^{1},\sigma_{i}^{1}\right)$ and $\left(\mu_{i}^{0},\sigma_{i}^{0}\right)$ for genes i=1,2,…,m under the alternative and null models, respectively. These parameters are estimated from the data on a gene-by-gene basis using maximum likelihood, which can be calculated by a least squares fit in the Normal distribution case. A clustering algorithm then assigns genes to k=1,2,…,K modules based on the symmetric Kullback–Leibler distance *δ_ik_* (only the alternative model is used to determine gene-module assignments). Using the module assignments, the parameter estimates are updated and genes are reassigned to new modules; the above steps continue until a convergence criterion is met. The final module parameters are denoted by $\left(c_{k}^{1},\upsilon_{k}^{1}\right)$ and $\left(c_{k}^{0},\upsilon_{k}^{0}\right)$ under the alternative and null models, respectively (Algorithm 1).


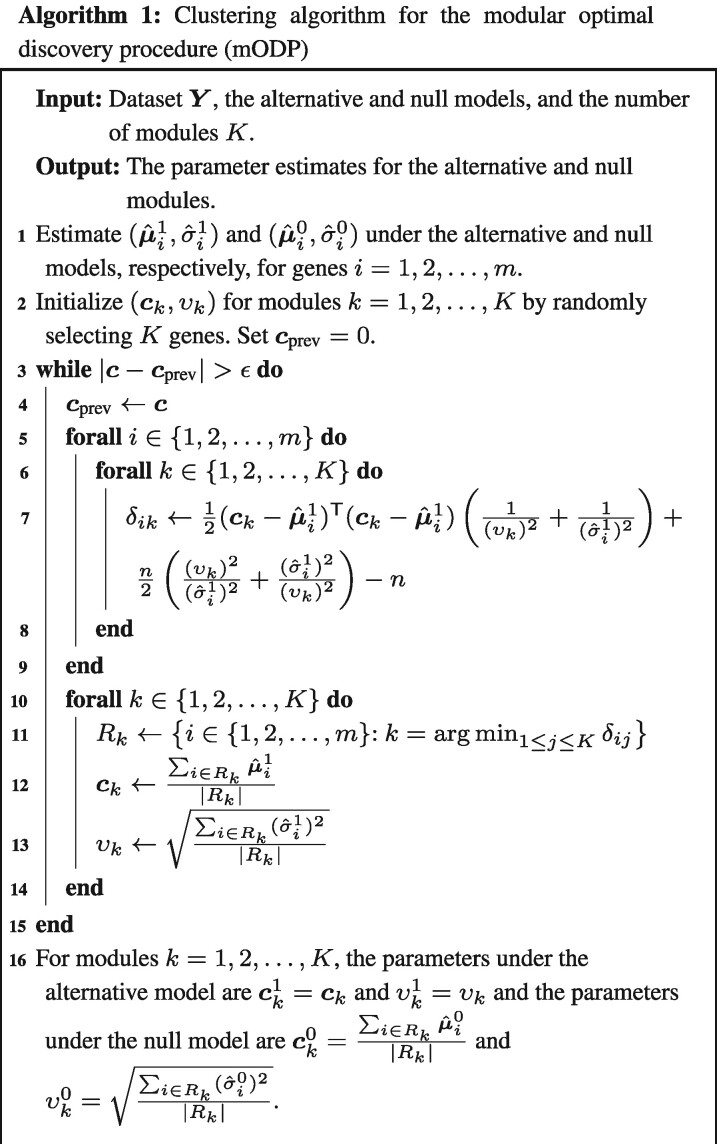


Given the module parameters, the mODP test statistic can be expressed as

(2) $${\hat{S}}_{\text{mODP}}(y_{i})=\frac{\sum_{k=1}^{K}g_{k}(y_{i};c_{k}^{1},\upsilon_{k}^{1})|R_{k}|}{\sum_{k=1}^{K}f_{k}(y_{i};c_{k}^{0},\upsilon_{k}^{0})|R_{k}|},$$

where $g_{k}(\cdot )$ is the alternative likelihood and $f_{k}(\cdot )$ is the null likelihood under module *k*, and $\left|R_{k}\right|$ is the number of genes assigned to module *k*. A bootstrap algorithm is implemented to generate the empirical null distribution of the test statistics (described in Supplementary Material). The mODP reduces the number of calculations from $2m^{2}$ to 2 *Km* where K≪m. Thus, the time complexity of the mODP is linear with the number of genes. In the study by Woo *et al.* (2011), the authors demonstrate that the mODP has similar power to the estimated ODP and is robust to the number of modules when K≥50.

### 2.2 Regression splines

The general framework for modeling non-linear responses in complex study designs follows from the study by Storey (2005). Consider an experiment with gene expression measurements *y_ij_* and explanatory variables *x_j_* for i=1,2,…,m genes and j=1,2,…,n observations. In non-static (e.g., time course) studies, there can be multiple measurements of *x_j_* for observation *j*, denoted by *x_jt_* where $t=1,2,\ldots ,T_{j}$; for example, *x_jt_* can be multiple time points or different dosage levels for a particular observation. The observed expression for gene *i* can be modeled as

(3) $$y_{\mathrm{ijt}}=\mu_{i}(x_{jt})+\gamma_{ij}+\epsilon_{\mathrm{ijt}},$$

where $\mu_{i}(\cdot )$ is the population average curve, *γ_ij_* is the individual-specific random deviation from the population average curve, and *ϵ_ijt_* is a random error that follows a Normal distribution with mean zero and variance $\sigma_{i}^{2}$. Here, we assume that the individual-specific random deviations follow a Normal distribution with mean zero and variance $\tau_{i}^{2}$.

The population average curve can be flexibly modeled using a regression spline. A regression spline is a piecewise polynomial function continuous at *d* specified points (or ‘knots’). We only consider natural cubic splines, which are third-order polynomial functions that are linear beyond the boundary knots. In this case, the population average curve can be parameterized by a *d*-dimensional basis: $\mu_{i}(x)=\alpha_{i}1+s(x)\;\beta_{i}$ where 1is a vector of 1's, $x=(x_{jt})$ is theset of explanatory variables, $s(x)=(s_{1}(x),s_{2}(x),\ldots ,s_{d}(x))$ is a matrix of the explanatory variables evaluated on a prespecified *d*-dimensional basis, and the parameters *α_i_* and $\beta_{i}={\left(\beta {}_{i1},\beta {}_{i2},\ldots ,\beta {}_{id}\right)}^{T}$ are estimated by least squares on a gene-by-gene basis (described in more detail in Supplementary Material). The parametric model for $\mu_{i}(x)$ enables testing of parameters *α_i_* and $\beta_{i}$, which do not depend on specific *x_jt_*. This simplification allows for inferences of general sampling designs (Storey *et al.*, 2005). We apply this framework to a static sampling study, two independent sampling non-static studies, and a longitudinal sampling study.

**Fig. 1.**
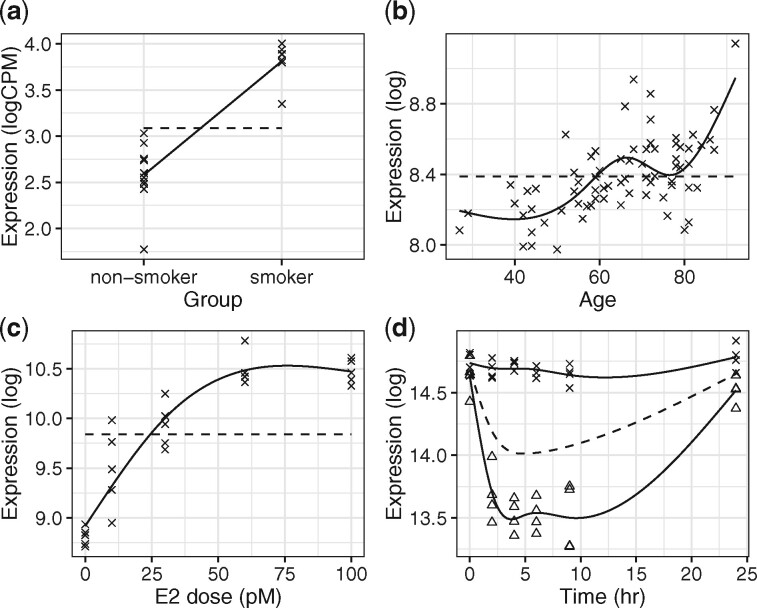
Fitting regression splines to general study designs: (**a**) static, (**b**, **c**) independent sampling and (**d**) longitudinal studies. The null (dashed) and alternative (solid) models are shown for a significant gene. In (d), the endotoxin-treated and control groups are denoted by a triangle and cross, respectively

In a static sampling study, subjects are independently sampled across one or more biological groups with no functional (e.g., time) component. As an example, in the smoker study, *x_j_* is a dichotomous variable taking values 0 or 1 indicating the smoking status of individual *j* and *y_ij_* is the RNA-seq count for gene *i* (Fig. 1a). Equation (3) can be simplified by modeling the population average curve as $\mu_{i}(x)=\alpha_{i}1+\beta_{i}x$ for the dichotomous variable $x={\left(x{}_{1},x{}_{2},\ldots ,x{}_{n}\right)}^{T}$ where the *t* index is dropped because implicitly *T_j_* = 1. In this study, we are interested in determining whether gene expression is differentially expressed between groups (the alternative hypothesis) or remains unchanged (the null hypothesis). Therefore, the null hypothesis model (dashed line) is fit under the constraint of $\beta_{i}=0$ and the alternative hypothesis model (solid line) allows this parameter to be unconstrained.

In an independent sampling study, subjects are independently sampled across a continuous variable (similar to cross-sectional sampling). The observed population average curve can be expressed as $\mu_{i}(x)=\alpha_{i}1+s(x)\beta_{i}$ for the continuous variable $x={\left(x{}_{1},x{}_{2},\ldots ,x{}_{n}\right)}^{T}$ where *T_j_* = 1. There are two studies analyzed with independent sampling designs. The first is a kidney aging study where human subjects are independently sampled at various ages (Fig. 1b). The second is a dose–response study where 17β-estradiol is introduced to breast cancer cells at various dosage levels (Fig. 1c). In both of these studies, the objective is to determine whether gene expression is differentially expressed across time or dosage level (the alternative hypothesis) or remains unchanged (the null hypothesis). Therefore, the null hypothesis model (dashed line) is fit under the constraint of $\beta_{il}=0$ for l=1,2,…,d and the alternative hypothesis model (solid line) allows these parameters to be unconstrained.

In a longitudinal sampling study, subjects are sampled multiple times across a continuous variable. Here, the observed population average curve can be expressed as $\mu_{i}(x)=\alpha_{i}1+s(x)\beta_{i}$ for the continuous variable $x=(x_{jt})$ where there are j=1,2,…,n observations and $t=1,2,\ldots ,T_{j}$ measurements of the *j*th observation. As an example, the endotoxin study compares two different classes across time, namely, endotoxin-treated versus control-treated. For this case, *y_ijt_* is the gene expression measurement for gene *i* in individual *j* at time point *t* and *x_jt_* indicates the time individual *j* was sampled (Fig. 1d). The alternative hypothesis is that there is differential expression between classes while the null hypothesis is that there is no difference in gene expression. Thus, the null hypothesis model fits one curve to both classes combined (dashed line) and the alternative hypothesis model fits two separate curves to each class (solid line).


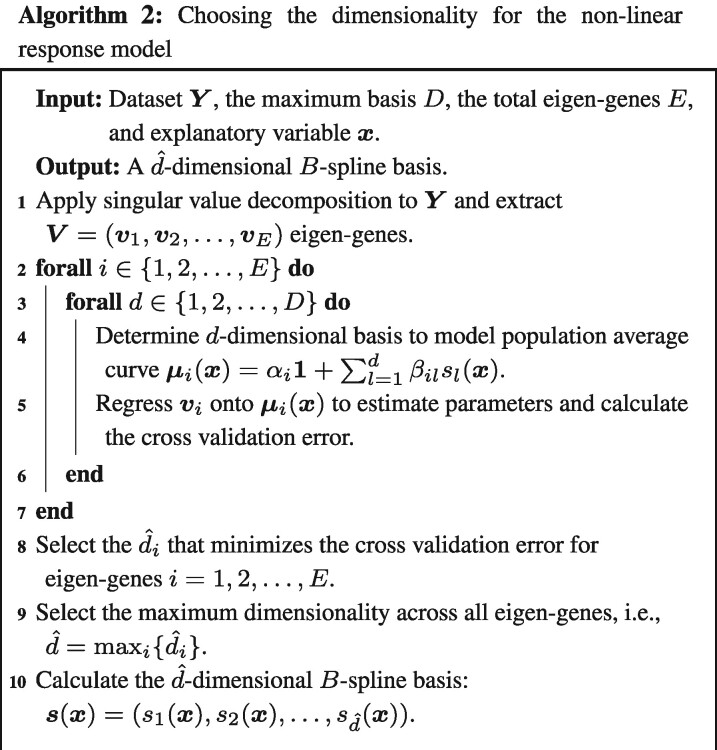


Algorithm 2 summarizes the procedure for choosing the dimensionality of the regression spline. The natural cubic spline—or any polynomial spline—can be parameterized by a *B*-spline basis. The *B*-spline requires choosing the location of *d* knots to anchor the basis functions. We utilize the cross validation algorithm by Storey *et al.* (2005) to automatically choose the optimal $\hat{d}$. First, we apply a singular value decomposition to extract the top i=1,2,…,E eigen-genes (or right-singular vectors) which represent directions of maximal variation. The eigen-genes are used for choosing a reasonable dimensionality to model the non-linear gene expression responses. We then regress these eigen-genes onto $s(x)=(s_{1}(x),s_{2}(x),\ldots ,s_{d}(x))$, where we use a d=1,2,…,D dimensional *B*-spline bases. The knots are placed at evenly spaced quantiles, i.e., the $0,\frac{1}{d-1},\frac{2}{d-1},\ldots ,\frac{d-2}{d-1},1$ quantiles. Finally, the basis dimension used for model fitting is chosen by applying a cross-validation procedure to select the an estimated optimal $\hat{d}$ across all eigen-genes. Using the selected $\hat{d}$, a least squares model fit is applied to estimate the population average curve and variance for all genes under the alternative and null models.

## 3 Materials and methods

Our algorithm introduces regression splines into the ODP framework to extend it to complex study designs. To do so, the ODP test statistic must be extended to incorporate non-linear responses. Suppose the expression values are *y_ijt_* and the explanatory variables are *x_jt_* where there are i=1,2,…,m genes, j=1,2,…,n observations and $t=1,2,\ldots ,T_{j}$ measurements of the *j*th observation. Consider two different models, namely, the null model with parameters $\left(\mu_{i}^{0}(x),\sigma_{i}^{0}\right)$ and the alternative model with parameters $\left(\mu_{i}^{1}(x),\sigma_{i}^{1}\right)$, where the null model is a restricted version of the alternative model (detailed in Supplementary Material). The objective is to test the null hypothesis $H_{0}:\mu_{i}(x)=\mu_{i}^{0}(x)$ versus the alternative hypothesis $H_{1}:\mu_{i}(x)=\mu_{i}^{1}(x)$. In this work, the population average curves are flexibly modeled using a regression spline. The parameters under both models can then be estimated by least squares, i.e., $\left({\hat{\mu }}_{i}^{1}(x),{\hat{\sigma }}_{i}^{1}\right)$ and $\left({\hat{\mu }}_{i}^{0}(x),{\hat{\sigma }}_{i}^{0}\right)$.


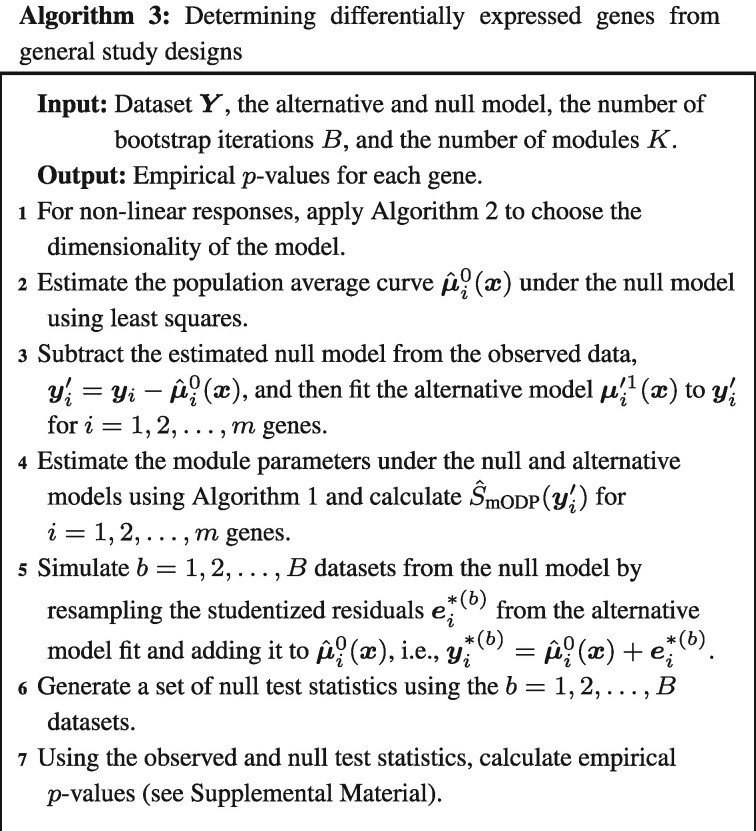


For non-linear responses, the estimated ODP statistic is

(4) $${\hat{S}}_{\text{ODP}}(y_{i})=\frac{\sum_{k=1}^{m}g_{k}(y_{i};{\hat{\mu }}_{k}^{1}(x),{\hat{\sigma }}_{k}^{1})}{\sum_{k=1}^{m}f_{k}(y_{i};{\hat{\mu }}_{k}^{0}(x),{\hat{\sigma }}_{k}^{0})},$$

where the likelihoods are assumed to follow a Normal distribution. It is evident that ${\hat{\mu }}_{i}^{0}(x)$ is not of interest in the testing procedure. This ancillary information can be removed by transforming the data to $y_{i}^{\prime }=y_{i}-{\hat{\mu }}_{i}^{0}(x)$. Under this transformation, the hypotheses are $H_{0}:\mu_{i}^{`1}(x)=0$ versus $H_{1}:\mu_{i}^{`1}(x)\neq 0$. This modified version of the estimated ODP statistic is

(5) $${\hat{S}}_{\text{ODP}}(y_{i}^{`})=\frac{\sum_{k=1}^{m}g_{k}(y_{i}^{`};{\hat{\mu }}_{k}^{`1}(x),{\hat{\sigma }}_{k}^{1})}{\sum_{k=1}^{m}f_{k}(y_{i}^{`};0,{\hat{\sigma }}_{k}^{0})}.$$

Similar to the original implementation of the ODP, the above test statistic requires $2m^{2}$ calculations which makes it computationally slow for genomic datasets. Instead, we can utilize the mODP statistic according to

(6) $${\hat{S}}_{\text{mODP}}(y_{i}^{`})=\frac{\sum_{k=1}^{K}g_{k}(y_{i}^{`};c_{k}^{1}(x),\upsilon_{k}^{1})|R_{k}|}{\sum_{k=1}^{K}f_{k}(y_{i}^{`};0,\upsilon_{k}^{0})|R_{k}|},$$

where there are k=1,2,…,K modules, the membership size of module *k* is $\left|R_{k}\right|$ and the module parameters are estimated by applying the mODP clustering algorithm (described in Algorithm 1).

Our proposed method is summarized in Algorithm 3: The inputs are the observed dataset (***x***,***Y***), the alternative and null models, the number of modules *K* and the number of bootstrap iterations *B*. First, we apply Algorithm 2 to choose the dimensionality *d* for the population average curves, i.e., $\mu_{i}(x)=\alpha_{i}1+s(x)\beta_{i}$ where $s(x)=(s_{1}(x),s_{2}(x),\ldots ,s_{d}(x))$. The data are then transformed by subtracting the null model fit from the observed dataset. This adjusted gene expression response variable $y_{i}^{\prime }$ is regressed onto the alternative model to get updated parameter estimates of $\left(\mu_{i}^{\prime 1}(x),\sigma_{i}^{1}\right)$. Finally, we apply the mODP clustering algorithm to determine the parameters $\left(c_{k}^{1}(x),\upsilon_{k}^{1}\right)$ and $\left(0,\upsilon_{k}^{0}\right)$ for the k=1,2,…,K modules under the alternative and null models, respectively (see Algorithm 1). Using the parameter estimates from the clustering algorithm, the mODP statistic is calculated for all genes. A bootstrap algorithm is implemented to calculate the empirical null distribution of the test statistics (described in Supplementary Material). For the datasets analyzed here, there are *B *=* *500 bootstrap iterations (*B *=* *5000 for the smoker study) and *K *=* *800 modules.

Prior applications of ODP have focused on microarray studies where it is common to assume that the gene expression response variable is approximately Normal and homoscedastic (i.e., a single variance per gene). However, in sequencing studies, this assumption is no longer valid because the observations are heteroscedastic. To apply the ODP to sequencing studies, we implement a similar strategy detailed by Law *et al.* (2014) where RNA-seq data are log-transformed to model the observed mean–variance relationship. Using this model, weights capturing the heteroscedasticity across observations are estimated. These weights are then incorporated in a weighted least squares regression and are easily integrated into the mODP framework. Given a set of inverse variance weights *w_ijt_* for i=1,2,…,m genes, j=1,2,…,n observations and $t=1,2,\ldots ,T_{j}$ measurements of the *j*th observation, the data are transformed as ${\bar{y}}_{\mathrm{ijt}}=\sqrt{w_{\mathrm{ijt}}}y_{\mathrm{ijt}}$ and ${\bar{s}}_{i}(x_{jt})=\sqrt{w_{\mathrm{ijt}}}s(x_{jt})$. An ordinary least squares algorithm can then be applied to this transformed data. Thus, Algorithm 3 can be appropriately adjusted to accommodate these weights.

## 4 Results

The generalized mODP was applied to four different genomic experiments. The performance of mODP was compared to an *F*-test and a moderated *F*-test (Smyth, 2004) using the number of discoveries and enriched gene sets. Finally, we validated our findings through comprehensive simulations.

### 4.1 Datasets

*Kidney study*. To elucidate the transcriptional response from aging in the kidney, the kidney study collected cortex samples from 72 patients with ages ranging from 27 to 92 years (Rodwell *et al.*, 2004). The samples were hybridized onto U133a and U133b GeneChips with 44 928 probes. Following similar filtering steps in Storey (2005) to control for potential confounding, only 38 833 probes were used for analysis and the expression values were log-transformed for variance stabilization.

*Endotoxin study*. The endotoxin study analyzed transcriptional regulation in human blood leukocytes from two experimental groups: a treatment group receiving a bacterial endotoxin (an inflammatory stimulus) and a control group (Calvano *et al.*, 2005). There were four samples in each biological group and blood samples were collected at 2, 4, 6, 9 and 24 h intervals. One control sample had missing information at the 4 and 6 h time points. The samples were hybridized onto U133 GeneChips with 44 924 probes. The expression values were log-transformed for variance stabilization.

*Smoker study*. The smoker study is a two group comparison between smoking and non-smoking humans (Ryan *et al.*, 2014). There are a total of 17 samples (10 non-smokers and 7 smokers) from human airway basal cells in the epithelium: there is one female smoker and the rest of the samples are males. The samples are sequenced (paired-end) using Illumina HiSeq 2000, and the reads are assembled using Bowtie: there are total of 65 217 genes with mapped reads. After filtering genes with fewer than 10 reads across all samples, only 26 268 genes remained for analysis. The R package limma is used to estimate the inverse-variance weights for the weighted least squares implementation. The expression values were transformed to log2-counts per million (logCPM).

*Dose study*. The dose study is a dose–response experiment where sensitivity to 17β-estradiol (E2) in breast cancer cells (BUS cells) was examined (Coser *et al.*, 2003). There are five biological replicates for each E2 concentration, where the E2 concentrations considered were 0, 10, 30, 60 and 100 pM (25 total samples). After 48 h exposed to E2, RNA samples were hybridized onto U133a GeneChips with 22 283 probes. The expression values were log-transformed to stabilize the variance.

### 4.2 Determining the degrees of freedom

**Fig. 2.**
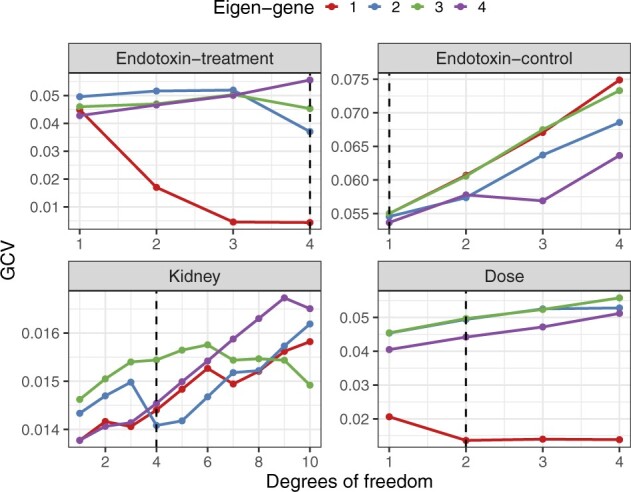
Cross-validation error versus degrees of freedom for the first four eigen-genes. The dotted line indicates the chosen degrees of the freedom in the study

We implemented the cross-validation procedure detailed in Algorithm 2 to determine the appropriate dimensionality *d* for the *B*-spline basis. (The smoker study is a two-group comparison and so regression splines are not necessary.) For each study, the first four eigen-genes were determined by applying a singular value decomposition to the dataset. In the endotoxin study, the control-treated and endotoxin-treated groups were separated into two distinct datasets. Multiple regressions were fit to the eigen-genes using *d =* 1, 2, 3, 4 for the endotoxin and dose studies and d=1,2,…,10 for the kidney study (an intercept term was included in the models). For each eigen-gene, the *d* that minimized the leave-one-out cross-validation error was selected. Finally, the maximum *d* across all eigen-genes was chosen as the estimated degrees of freedom. Applying the above procedure, we find $\hat{d}=4$ for the endotoxin and kidney studies and $\hat{d}=2$ for the dose study (Fig. 2).

### 4.3 Method comparisons

We compared the mODP to two other popular test statistics, namely, the *F*-statistic and the moderated *F*-statistic (described in Supplementary Material). Compared to the *F*-statistic, the moderated *F*-statistic shrinks the sample variance toward a pooled variance. This shrinkage allows for more stable inferences with low-sample size studies (Smyth, 2004). Unlike the mODP which requires an empirical null distribution, the *F*-statistic and moderated *F*-statistic have theoretical null distributions. Therefore, we also estimate an empirical null distribution for the *F*-test and moderated *F*-test using a bootstrap algorithm (described in Supplementary Material). In summary, the mODP is compared to an *F*-test, a moderated *F*-test, a bootstrap *F*-test and a bootstrap moderated *F*-test.

**Fig. 3.**
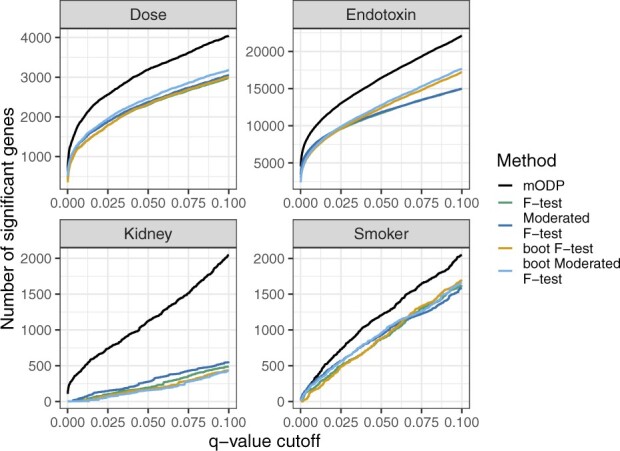
Observed number of discoveries at various *q*-value cutoffs from the mODP (black), *F*-test (green), bootstrap *F*-test (orange), moderated *F*-test (blue) and bootstrap moderated *F*-test (light blue). These methods were applied to four different studies: endotoxin (longitudinal sampling), kidney (independent sampling), dose (independent sampling) and smoker (static sampling)

**Fig. 4.**
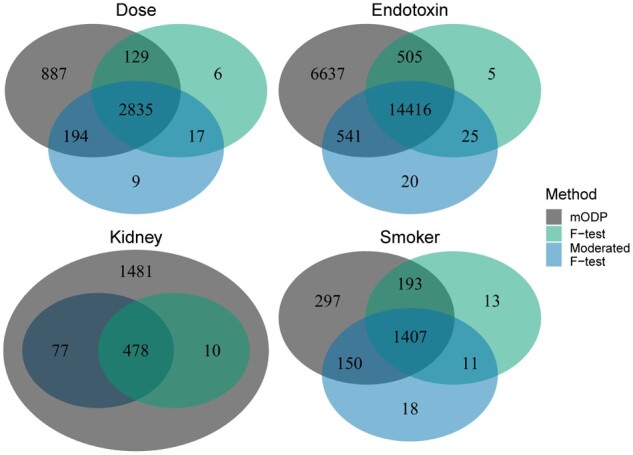
Venn diagram of the total discoveries at a FDR of 0.1 for the studies in Figure 3. Only the mODP (black), *F*-test (green) and moderated *F*-test (blue) are shown

We applied the above testing procedures to our four chosen studies and calculated the number of differentially expressed genes at various FDRs (Fig. 3). At each FDR threshold, the mODP finds substantially more differentially expressed genes compared to the other methods. For example, when applying a FDR of 0.1, mODP detects 1481, 297, 6637 and 887 more differentially expressed genes in the kidney, smoker, endotoxin and dose studies, respectively. In addition, the mODP finds nearly all of the differentially expressed genes detected by the other methods (Fig. 4). Finally, we find that the mODP has the lowest estimated proportion of true nulls across all studies (Supplementary Table S1). This suggests that the mODP estimates a higher expected number of alternative genes.

**Fig. 5.**
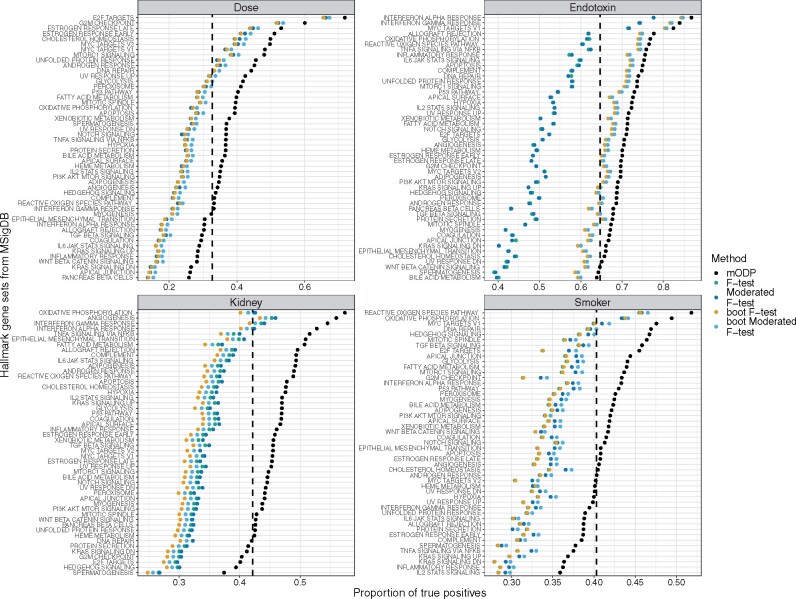
Proportion of true positives for the Hallmark gene sets from MSigDB. Enrichment results for each method are shown for the dose, endotoxin, kidney and smoker studies. The dashed line indicates the proportion of true positives in the dataset using mODP

To compare the testing procedures, we also performed an enrichment analysis using the hallmark gene sets from the MSigDB database. These gene sets contain highly curated genes with clear expression for well-defined biological states or processes (Liberzon *et al.*, 2015, 2011). We developed a simple procedure to detect important gene sets by assigning the proportion of true positives to each. These values range from 0 to 1, with important gene sets having largest values (see Supplementary Material for additional details). We find that the mODP has the largest proportion of true positives across all gene sets compared to other methods (Fig. 5). Thus the mODP has more power to detect gene sets with enriched true positives.

### 4.4 Simulations

Comprehensive simulations were performed to verify the observed differences between mODP and other methods. We generated 500 representative datasets of the observed studies as follows. For each study, the *F*-testing procedure was used to separate genes into two distinct classes (alternative and null) based on a FDR threshold of 0.1. We then sampled from the population of alternative genes to get unique gene expression profiles. In total, we considered 5, 10, 50, 100 and 200 unique gene expression curves in our simulation studies. These curves defined the signal for the alternative genes. (Note the smoker study is a static experiment and so ‘unique gene expression profile’ refers to the mean differences between the two conditions.) The signals for the null genes were sampled from the null population. Random noise was added to maintain the signal-to-noise ratio and to match the observed power. Finally, the total number of alternative and null genes was chosen to keep the observed proportion of true nulls fixed. For more details, see Supplementary Material.

**Fig. 6.**
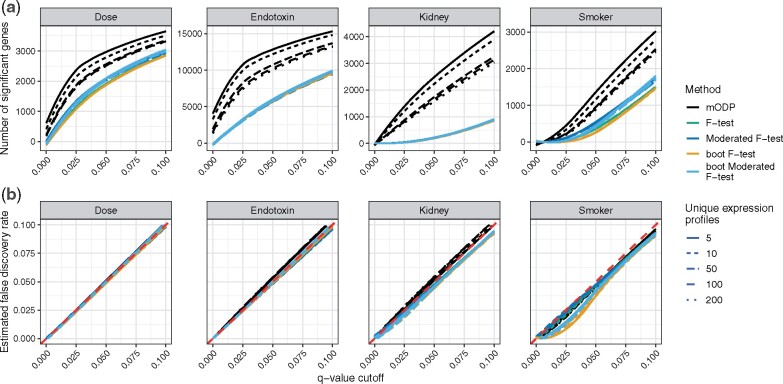
Simulation results for the dose, endotoxin, kidney and smoker studies using 5, 10, 50, 100 and 200 unique gene expression profiles (linetype). The mODP (black), *F*-test (green), moderated *F*-test (blue), bootstrap *F*-test (orange) and bootstrap moderated *F*-test (light blue) are applied to the simulated studies. (**a**) Estimated power at multiple *q*-value cutoffs between 0.0001 and 0.1. (**b**) Estimated FDR. Curves represent the average value from 500 replications

To compare the testing procedures in the simulated datasets, we calculated the estimated FDR and the total number of discoveries. We find that the mODP controls the FDR in all simulated studies (Fig. 6b). Furthermore, substantially more differentially expressed genes were detected relative to the other testing procedures (Fig. 6a). We also find that the moderated *F*-test identifies a similar number of differentially expressed genes compared to the *F*-test. This is unsurprising as the moderated *F*-test only outperforms the *F*-test when there are small sample sizes. When the number of unique gene expression patterns is increased, the power of the mODP decreases while the *F*-test and moderated *F*-test remain unchanged. This occurs because the number of unique gene expression curves does not change the power of the *F*-test and moderated *F*-test.

### 4.5 Implementation in the edge package

The mODP described above is implemented in an R package called edge. When applying the edge package to a dataset, the first step is to formulate an alternative and null hypothesis to test. Our software has a function called build_study to help users formulate hypotheses for static, longitudinal or independent sampling designs. Once the two hypotheses are specified, the next step is to input the design matrices into the odp function to apply the mODP procedure outlined here. (Note that the clustering and bootstrap algorithms are executed internally with default parameters that can be changed.) The odp function returns significance results such as *P*-values, local FDRs, *q*-values, and the proportion of truly null tests. These results can then be visualized using the plot and hist functions in a statistical analysis.

There are many other features included in our software: (i) the gene-module assignments are available for additional analyses (e.g. enrichment analysis) using the kl_clust function, (ii) the model fits under the alternative and null hypotheses can be accessed using the fit_models function and (iii) integrations of other popular tools such as surrogate variable analysis (SVA) (Leek and Storey, 2007, 2008) and jackstraw (Chung and Storey, 2015) are available for more comprehensive biological analyses. Additional information can be found in the vignette at http://bioconductor.org/packages/release/bioc/html/edge.html.

## 5 Discussion

The ODP is a test statistic that provides substantial improvements in statistical power compared to other testing procedures. While previous work on the ODP is limited to static microarray studies (Storey *et al.*, 2007; Storey, 2007; Woo *et al.*, 2011), here, we extend its application to complex experimental designs and sequencing studies. Our proposed algorithm is applied to two time-series studies, a dose–response study and an RNA-seq study. For each study, our method detects more differentially expressed genes and improves the statistical power for gene set enrichment analysis. These improvements in power are validated through comprehensive simulations, where data are simulated to closely resemble the observed datasets. Therefore, the ODP allows for a more thorough investigation of underlying biological mechanisms in downstream analysis.

The gained improvements in power from the ODP have important biological implications. In a genome-wide gene expression study, genes are commonly co-expressed and share similar patterns of gene expression. The ODP leverages this information across genes to strengthen the evidence for or against differential expression. To explore how the ODP depends on the ‘degree’ of co-expression, we varied the number of unique gene expression profiles with simulated data. We find that the ODP loses power as the number of unique gene expression profiles increases; there are fewer related genes and so there is less information that can be leveraged in the test statistic. As an extreme case, suppose genes are not co-expressed and every gene follows a unique expression pattern. In this scenario, the ODP has been shown to perform similar to the *F*-test (Storey *et al.*, 2007). While this example is unlikely in biological studies, it provides intuition for the observed power improvements compared to other testing procedures.

There are a few considerations to note when applying our extended framework to genomic datasets. First, the computationally efficient implementation of the ODP, called the modular ODP (mODP), involves specifying the number of modules. While previous work has recommended 50 modules for static microarray studies, we found choosing at least 200 modules to capture the complex relationships among genes provides the best results. Second, there needs to be an adequate number of observations in the study. This is due to the constraints of the mODP: it requires accurate estimates of the mean and variance. Furthermore, the bootstrap algorithm implemented in the procedure requires a minimum number of observations per biological condition to generate a valid empirical null. In the studies considered here, there are at least four biological replicates per condition. Finally, the appropriate degrees of freedom need to be carefully chosen to avoid overfitting the spline. To this end, we implemented a procedure from the study by Storey *et al.* (2005) that chooses the degrees of freedom based on the leave-one-out cross validation algorithm.

An interesting aspect of the mODP implementation is that a clustering algorithm assigns genes to modules, where the modules are representative of shared gene expression patterns. These modules provide valuable information that can be utilized in an exploratory data analysis. For example, we can calculate the proportion of true positives for each module and rank modules based on true-positive enrichment. Modules enriched with true positives can then be further analyzed to understand functional relationships among genes. Thus the clustering algorithm provides information of potential use in other biological analyses.

There are a number of ways the ODP can be further extended for genomic studies. One enhancement is incorporating prior weights on each hypothesis test. For example in sequencing data, higher per-gene read counts are more reliable than lower per-gene read counts. This information can be included into a weighted ODP, where weights are generated by estimating the functional proportion of true nulls based on some informative variable (Chen *et al.*, 2017). Another enhancement is to extend the ODP to generalized linear models where the response variable follows an exponential family distribution.

As the cost of generating biological samples decreases, the prevalence of complex study designs will increase. The key motivation behind these studies is to capture inherently non-linear transcriptional responses. Therefore, there is demand for statistically rigorous methodologies that can be applied to such settings. In this work, we develop a framework to model non-linear gene expression responses while optimally utilizing biological correlations among genes to improve statistical power. Our method can thus uncover important biological insights across a wide range of applications in functional, translational and clinical genomics.

## 6 Software and data

An implementation of the algorithm described in this article is available as an R package called edge. The package can be downloaded at https://github.com/StoreyLab/edge (most recent version) or https://bioconductor.org/packages/release/bioc/html/edge.html (release). The data and code used to produce the figures in this manuscript can be found at https://github.com/StoreyLab/odp_general_studies.

## Funding

This work was supported by NIH grants R01 HG002913, R01 HG006448, and 5T32HG003284. 

*Conflict of Interest*: none declared.

## Supplementary Material

btaa707_Supplementary_Data
