# Supplementary material for: The optimal discovery procedure for significance analysis of general gene expression studies
Source: Bioinformatics. 2020 Aug 20;37(3):367–74. doi: 10.1093/bioinformatics/btaa707 (PMC8058779; doi:10.1093/bioinformatics/btaa707)
Supplement: btaa707_Supplementary_Data [file btaa707_supplementary_data.pdf]

Supplementary Material in *Bioinformatics*: The optimal discovery procedure for significance analysis of general gene expression studies

Andrew J. Bass and John D. Storey*

Lewis-Sigler Institute for Integrative Genomics
Princeton University
Princeton, NJ 08544 USA

1 The F-statistic and moderated F-statistic

Suppose gene expression data y_{ijt} and explanatory variables x_{jt} are observed for $i = 1, 2, \ldots, m$ genes, $j = 1, 2, \ldots, n$ observations, and $t = 1, 2, \ldots, T_j$ measurements of the jth observation. The most general model we consider in this paper is

$$y_{ijt} = \mu_i(x_{jt}) + \gamma_{ij} + \epsilon_{ijt}, \tag{1}$$

where $\mu_i(\cdot)$ is the population average mean function of explanatory variables x_{jt}, γ_{ij} is an individual-specific random deviation, and $\epsilon_{ijt} \overset{iid}{\sim}$ Normal$(0, \sigma_i^2)$. (In the case of RNA-seq data, we may model the heteroscedastic variance as $\frac{1}{w_{ijt}}\sigma_i^2$ as discussed in the main paper.) For the case that $T_j = 1$ for all $j = 1, 2, \ldots, n$, this model reduces to

$$y_{ij} = \mu_i(x_j) + \epsilon_{ij}, \tag{2}$$

where γ_{ij} has been implicitly absorbed into ϵ_{ij}.

In dealing with the case that $T_j > 1$, see [1] for more details. Fitting the model, forming test statistics, and performing bootstrap sampling is much more complex in this scenario, and it is the main focus of [1]. Here, we provide details for the simpler case that $T_j = 1$ for all $j = 1, \ldots, n$. We model $\mu_i(\cdot)$ according to a d-dimensional set of basis functions, where $\mu_i(x_j) = \alpha_i + \sum_{l=1}^{d} \beta_l s_l(x_j)$. We can write this in vector notation. Let $\boldsymbol{s}(\boldsymbol{x}) = (s_1(\boldsymbol{x}), s_2(\boldsymbol{x}), \ldots, s_d(\boldsymbol{x}))$ be the $n \times d$ design matrix with entry

*Corresponding author: jstorey@princeton.edu

(jl) equal to $s_l(x_j)$. The matrix $\boldsymbol{s}(\boldsymbol{x})$ is assumed to be full rank. Also, let $\mathbf{1}$ be an n-vector composed of 1's. The model is then

$$\begin{aligned}\boldsymbol{y}_i &= \alpha_i \mathbf{1} + \boldsymbol{s}(\boldsymbol{x})\boldsymbol{\beta}_i^T + \boldsymbol{\epsilon}_i \\ &= \alpha_i \mathbf{1} + \sum_{l=1}^{d} \beta_{il} s_l(\boldsymbol{x}) + \boldsymbol{\epsilon}_i\end{aligned} \tag{3}$$

where, for example, $\boldsymbol{y}_i = (y_{i1}, y_{i2}, \ldots, y_{in})^T$.

In this case, the null model $\boldsymbol{y}_i = \alpha_i \mathbf{1} + \sum_{l=1}^{d_0} \beta_{il} s_l(\boldsymbol{x}) + \boldsymbol{\epsilon}_i$ is tested versus the alternative model $\boldsymbol{y}_i = \alpha_i \mathbf{1} + \sum_{l=1}^{d} \beta_{il} s_l(\boldsymbol{x}) + \boldsymbol{\epsilon}_i$ where $1 \leq d_0 < d$ and $\boldsymbol{\epsilon}_i$ are uncorrelated random errors that follow a Normal distribution with mean zero and variance σ_i^2. We are interested in comparing both models to infer whether $\beta_{il} \neq 0$ for at least one of the $l = d_0 + 1, \ldots, d$ explanatory variables.

Note that a standard linear model can be written as a special case of the above model. Consider a standard $n \times d$ design matrix $\boldsymbol{x}$ composed of d explanatory variables over n observations. Suppose we also define $s_l(\boldsymbol{x}) = \boldsymbol{x}_l$, where $\boldsymbol{x}_l$ is the lth column of $\boldsymbol{x}$ corresponding to the n observed values of explanatory variable l. The above formulation then translates to the model $\boldsymbol{y}_i = \alpha_i \mathbf{1} + \sum_{l=1}^{d} \beta_{il} \boldsymbol{x}_l + \boldsymbol{\epsilon}_i$, which is a standard linear model.

The F-test is a classical testing procedure that can be used to compare nested regression models. The general procedure works as follows. The alternative model is fit using least squares to the observed data to estimate the parameters $(\hat{\alpha}_i, \hat{\boldsymbol{\beta}}_i)$ and the residual vector $\boldsymbol{e}_i = \boldsymbol{y}_i - \hat{\alpha}_i \mathbf{1} - \sum_{l=1}^{d} \hat{\beta}_{il} s_l(\boldsymbol{x})$. Similarly, the null model is fit to estimate the parameters $(\hat{\alpha}_i, \hat{\boldsymbol{\beta}}_i^{\text{null}})$ and the residual vector $\boldsymbol{e}_i^{\text{null}} = \boldsymbol{y}_i - \hat{\alpha}_i \mathbf{1} - \sum_{l=1}^{d_0} \hat{\beta}_{il}^{\text{null}} s_l(\boldsymbol{x})$. The test statistic is defined as

$$F_i = \frac{\left(\left\|\boldsymbol{e}_i^{\text{null}}\right\|^2 - \left\|\boldsymbol{e}_i\right\|^2\right)/(d - d_0)}{\left\|\boldsymbol{e}_i\right\|^2/(n - d - 1)}, \tag{4}$$

where the theoretical distribution under the null hypothesis follows Fisher's F-distribution with $d - d_0$ degrees of freedom in the numerator and $n - d - 1$ degrees of freedom denominator (denoted $F_{d-d_0, n-d-1}$). Intuitively, if there is no difference between both models then F_i should be concentrated around 1. Otherwise, large deviations from 1 provide evidence against the null model. The assumption that the F-statistic follows an F-distribution under the null hypothesis is only true asymptotically: in practice, large sample sizes are necessary for reliable inferences.

For small sample sizes, the moderated F-statistic can be used to compare two models. The main issue with small sample sizes is that the sample variance can often be inflated and unreliable to use in the traditional F-test. The moderated F-test is an empirical Bayes procedure that borrows information across genes to provide stable estimates of the sample variance [2]. A rough outline of the hierarchical model is as follows. The inverse variance across genes are assumed to vary as a scaled chi-square

distribution, i.e.,

$$\frac{1}{\sigma_i^2} \sim \frac{1}{\rho_0 \sigma_0^2} \chi^2_{\rho_0}, \tag{5}$$

where ρ_0 is the degrees of freedom and σ_0^2 is a scaling factor. Furthermore, the non-zero effect sizes are assumed to follow a Normal distribution with mean zero and variance proportional to σ_i^2. The posterior mean of σ_i^2 given the sample variance can be determined from the above hierarchical model, see [2] for more details. This mean value is used as an improved estimate of the sample variances, where the sample variances are shrunken towards the prior estimator σ_0^2 for more stable estimates. More specifically, the moderated F-statistic is defined as

$$F_i = \frac{\left(\left\|\boldsymbol{e}_i^{\text{null}}\right\|^2 - \left\|\boldsymbol{e}_i\right\|^2\right)/(d-d_0)}{\left\|\boldsymbol{e}_i^*\right\|^2/((n-d-1)+\rho_0)}, \tag{6}$$

where $\left\|\boldsymbol{e}_i^*\right\|^2 = \left\|\boldsymbol{e}_i\right\|^2 + \rho_0\sigma_0^2$ and the parameters (ρ_0, σ_0^2) are estimated from the data [2]. The theoretical distribution under the null hypothesis for the moderated F-statistic follows an F-distribution with $d-d_0$ degrees of freedom in the numerator and $(n-d-1)+\rho_0$ degrees of freedom in the denominator, $F_{d-d_0,(n-d-1)+\rho_0}$. For large sample sizes, the statistical power of the moderated F-test will be similar to the classical F-test.

2 Generating bootstrap empirical null statistics

A standard bootstrap procedure was implemented to generate an empirical null distribution for the testing procedures as follows.

1. Assume the null model is $\boldsymbol{y}_i = \boldsymbol{\mu}_0(\boldsymbol{x}) + \boldsymbol{\epsilon}_i$ and the alternative model is $\boldsymbol{y}_i = \boldsymbol{\mu}_1(\boldsymbol{x}) + \boldsymbol{\epsilon}_i$ where $\boldsymbol{\epsilon}_i$ are the random errors.

2. Fit both models to the observed data using least squares or weighted least squares (if per-observation weights are available). Estimate $\hat{\boldsymbol{\mu}}_1(\boldsymbol{x})$ for the alternative model and $\hat{\boldsymbol{\mu}}_0(\boldsymbol{x})$ for the null model. Calculate the test statistic of interest (i.e., the mODP statistic, F-statistic, or moderated F-statistic), denoted by $T_i(\hat{\boldsymbol{\mu}}_0(\boldsymbol{x}), \hat{\boldsymbol{\mu}}_1(\boldsymbol{x}))$ for genes $i = 1, 2, \ldots, m$.

3. For $b = 1, 2, \ldots, B$ bootstrap samples, sample n observations from the studentized residuals (with replacement) to obtain $\boldsymbol{e}_i^{*(b)}$. Add these residuals to the null model fit, i.e., $\boldsymbol{y}_i^{*(b)} = \hat{\boldsymbol{\mu}}_0(\boldsymbol{x}) + \boldsymbol{e}_i^{*(b)}$. If there are weights, the studentized residuals should be appropriately rescaled.

4. Fit both models to $\boldsymbol{y}_i^{*(b)}$ and obtain $\hat{\boldsymbol{\mu}}_0^{*(b)}(\boldsymbol{x})$ and $\hat{\boldsymbol{\mu}}_1^{*(b)}(\boldsymbol{x})$ estimates under the null and alternative models, respectively. Calculate $T_i^{*(b)}\left(\hat{\boldsymbol{\mu}}_0^{*(b)}(\boldsymbol{x}), \hat{\boldsymbol{\mu}}_1^{*(b)}(\boldsymbol{x})\right)$ for $b = 1, 2, \ldots, B$ bootstrap samples

and genes $i = 1, \ldots, m$. Note that the hyperparameters of the moderated F-statistic (d_0, σ_0) are fixed and so $\|\boldsymbol{e}_i^*\|^2 = \left\|\boldsymbol{e}_i^{*(b)}\right\|^2 + d_0\sigma_0^2$.

5. Calculate the empirical p-values according to

$$p_i = \frac{\sum_{a=1}^{m}\sum_{b=1}^{B} \mathbf{1}\left(T_a^{*(b)}(\hat{\boldsymbol{\mu}}_0^{*(b)}(\boldsymbol{x}), \hat{\boldsymbol{\mu}}_1^{*(b)}(\boldsymbol{x})) \geq T_i(\hat{\boldsymbol{\mu}}_0(\boldsymbol{x}), \hat{\boldsymbol{\mu}}_1(\boldsymbol{x}))\right)}{mB}.$$

When applying the mODP bootstrap, the above procedure requires a few modifications. First, the data is adjusted to $\boldsymbol{y}_i' = \boldsymbol{y}_i - \hat{\boldsymbol{\mu}}_i^0(\boldsymbol{x})$ in step (2) to remove ancillary information (i.e., $\hat{\boldsymbol{\mu}}_i^0(\boldsymbol{x})$). The alternative model is then fit to $\boldsymbol{y}_i'$. (Note that the design matrix from the alternative model is adjusted by $(\boldsymbol{I} - \boldsymbol{H}_{\text{null}})$ where $\boldsymbol{H}_{\text{null}}$ is the projection matrix under the null model. Additionally, if there are weights, $\boldsymbol{y}_i'$ and the design matrix are appropriately adjusted before fitting the alternative model.) Second, the studentized residuals are rescaled by the observed sample variance. This enforces that the sample variance remains the same for all bootstrap iterations. Thus the rescaled studentized residuals in step (3) are $\frac{\hat{\sigma}_i}{\hat{\sigma}_i^{*(b)}}\boldsymbol{e}_i^{*(b)}$ where $\hat{\sigma}_i = \frac{\|\boldsymbol{e}_i\|}{\sqrt{n-d-1}}$ is the sample standard deviation of the residuals from the alternative model and $\hat{\sigma}_i^{*(b)} = \frac{\left\|\boldsymbol{e}_i^{*(b)}\right\|}{\sqrt{n-1}}$ is the standard deviation from the resampled residuals. Finally, in step (4), the null statistics are recomputed with the module parameters estimated from the observed data.

It is important to note that additional steps in the above algorithm may need to be taken when handling longitudinal data. See ref. [1] for more details.

3 Simulation details

The primary objective in the simulations is to generate replicate datasets of the studies. We use the biological signal from each study as a baseline: both models are fit to estimate the gene expression curves under the alternative and null models. The genes assigned to the alternative model had q-values < 0.1 while genes assigned to the null model had q-values > 0.1. The number of unique curves from the alternative model was varied by randomly selecting from the population of genes assigned to the alternative model. For each study and number of unique gene expression curves $G = 5, 10, 50, 100, 200$, the procedure is outlined below:

1. Use the estimated proportion of true nulls $\hat{\pi}_0$ to randomly assign the m genes to either the alternative or null models. Genes assigned to the alternative model followed one of the $g = 1, 2, \ldots, G$ unique gene expression curves, i.e. $\boldsymbol{\mu}_1^g(\boldsymbol{x})$. Alternatively, the null genes were randomly sampled from the population of null model fits $\boldsymbol{\mu}_0^*(\boldsymbol{x})$.

	ODP	F-test	Mod. F-test	boot. F-test	boot. mod. F-test
Dose	0.672	0.803	0.813	0.804	0.793
Endotoxin	0.349	0.605	0.615	0.388	0.397
Kidney	0.585	0.687	0.685	0.684	0.676
Smoker	0.597	0.681	0.681	0.680	0.675

Table S1: Estimated proportion of true nulls.

2. Using the observed signal-to-noise ratio (SNR) distribution from the alternative model, calculate an appropriate SNR_M such that the estimated number of differential expressed genes at a false discovery rate of 0.1 is close to the observed study. This was done by trial and error: $\boldsymbol{y}_i = \boldsymbol{\mu}_1^g(\boldsymbol{x}) + \sigma_i^*$, where σ_i^* is randomly sampled from the population of standard deviations $\frac{\sigma_g}{\sqrt{\text{SNR}_\text{M}}}$ for $g = 1, 2, \ldots, G$.

3. Randomly sample from the population of standard deviations in the previous step to add noise to the alternative model $\boldsymbol{y}_i = \boldsymbol{\mu}_1^g(\boldsymbol{x}) + \sigma_i^*$ and the null model $\boldsymbol{y}_i = \boldsymbol{\mu}_0^*(\boldsymbol{x}) + \sigma_i^*$ for all genes; call this simulated dataset $\boldsymbol{Y}^*$.

4. Apply the testing procedures to $\boldsymbol{Y}^*$ and calculate p-values.

5. Repeat steps (3-4) 500 times and calculate the average number of discoveries and the average false discovery rate for all testing procedures.

The estimated proportion of true nulls are shown in Table 1 and the estimated SNR_M for the dose, endotoxin, kidney, and smoker studies are the 0.86, 0.45, 0.35, and 0.80 quantiles of the SNR distribution, respectively.

4 True positive enrichment

Consider $i = 1, 2, \ldots, m$ test statistics z_i calculated on a gene-by-gene basis from a biological study. Given these test statistics, we propose a new summary statistic for gene sets based on the proportion of true positives. The procedure works as follows. For each gene i, we can calculate the local false discovery rate based on the chosen test statistic:

$$\text{lfdr}(z_i) = \text{Pr}\{\text{null hypothesis } i \text{ true}|z_i\} = \pi_0 \frac{f_0(z_i)}{f(z_i)},$$

where π_0 is the prior probability that a hypothesis test is null, $f_0(z_i)$ is the null density, and $f(z_i)$ is a mixture of the null and alternative densities [3, 4]. Next, we average the local false discovery rate in

gene set S,

$$\Lambda(S) = \frac{1}{|S|} \sum_{i \in S} (1 - \text{lfdr}(z_i)).$$

As an example, if we calculated $\Lambda(S) = 0.9$ then it corresponds to a gene set with an average of 0.9 true positives. Thus this gene set has a high proportion of true positives. The true positive enrichment (TPE) can be defined as

$$\text{TPE}(S) = \frac{\sum_{i \in S}(1 - \text{lfdr}(z_i))}{|S|(1 - \pi_0)},$$

where TPE(S) compares the number of expected true positives in gene set S to a randomly assembled gene set of the same size. An equivalent interpretation is the ratio of the average number of true positives in set S to the average across all genes, i.e., TPE$(S) = \frac{\Lambda(S)}{1-\pi_0}$. (Note that the average number of true positives across all genes is $\frac{1}{m}\sum_{i=1}^{m}(1 - \text{lfdr}(z_i)) = 1 - \pi_0$). Here, the statistic $\Lambda(S)$ is used because we are comparing different testing procedures.

The advantages of working in this framework are (i) it is computationally fast to calculate $\Lambda(S)$ for all gene sets, (ii) the interpretation of important gene sets is intuitive, and (iii) covariate-adjusted local false discovery rates can easily be incorporated to improve statistical power.

References

[1] John D. Storey, W. Xiao, J. T. Leek, R. G. Tompkins, and R. W. Davis. Significance analysis of time course microarray experiments. *Proceedings of the National Academy of Sciences*, 102(36): 12837–12842, sep 2005. doi: 10.1073/pnas.0504609102. URL https://dx.doi.org/10.1073/pnas.0504609102.

[2] Gordon K Smyth. Linear models and empirical bayes methods for assessing differential expression in microarray experiments. *Statistical Applications in Genetics and Molecular Biology*, 3(1):1–25, 2004. doi: 10.2202/1544-6115.1027. URL https://www.degruyter.com/view/j/sagmb.2004.3.issue-1/sagmb.2004.3.1.1027/sagmb.2004.3.1.1027.xml.

[3] Bradley Efron, Robert Tibshirani, John D Storey, and Virginia Tusher. Empirical bayes analysis of a microarray experiment. *Journal of the American Statistical Association*, 96(456):1151–1160, 2001. doi: 10.1198/016214501753382129. URL https://doi.org/10.1198/016214501753382129.

[4] John D. Storey. The positive false discovery rate: A bayesian interpretation and the q-value. *Annals of Statistics*, 31(6):2013–2035, dec 2003. doi: 10.1214/aos/1074290335. URL https://dx.doi.org/10.1214/aos/1074290335.
